# Use of a 3D exoscope in microvascular decompression of the trigeminal nerve root

**DOI:** 10.3171/2023.10.FOCVID23149

**Published:** 2024-01-01

**Authors:** Johannes Herta, Karl Rössler, Christian Dorfer

**Affiliations:** Department of Neurosurgery, Medical University of Vienna, Austria

**Keywords:** microvascular decompression, trigeminal neuralgia, 3D exoscope

## Abstract

For microvascular decompression surgery, adequate visualization of the trigeminal nerve root is essential. Several visualization techniques with operating microscopes, endoscopes, a combination of both, and exoscopes have been described. In this video, the authors use a 4K 3D exoscope (ORBEYE) as it offers superb optical image quality with a high degree of magnification and illumination in the cerebellopontine angle. Other advantages are surgeon ergonomics, a very good depth of field for the entire operating team, and potentially evolving visualization technologies like narrow-band imaging—essential points for microvascular decompression surgery where the aim is to create the best possible visibility in a narrow corridor.

The video can be found here: https://stream.cadmore.media/r10.3171/2023.10.FOCVID23149

## Transcript

In this video we describe the use of a 3D exoscope and the technical nuances to perform vascular decompression of the trigeminal nerve root in patients suffering from trigeminal neuralgia.

### 0:34 Patient Positioning.

We prefer to operate on the patient in the prone position with the head turned and inclined by approximately 30°, as this allows us to position the patient very steeply. This in turn leads to a very good CSF drainage and a relaxed cerebellum without having to worry about air embolism.

Landmarks such as the asterion and the course of the transverse and sigmoid sinus are drawn.

### 1:07 Surgical Opening Technique.

A classic retrosigmoid approach is used. Neck muscles are cut, the occipital artery is cauterized if needed, and the periosteum is dissected from the bone with a raspatory.

Bone sutures are identified and the bone is removed over the asterion to identify the angle between the transverse and sigmoid sinus. Only a small craniotomy of approximately 2 cm in width and height is needed. Bone wax is used to close mastoid air cells as well as emissary vein canals. The dura is opened in a Y-shaped fashion between the sinuses. Sutures are attached to augment exposure.^1^

### 2:06 Exoscope Position.

The exoscope is positioned behind the surgeon with the 3D camera placed in front of the surgeon’s chest, which has been named a "cuddly position."^2,3^ This position has major advantages. Firstly, the microscope-like viewing angle makes orientation easier for a newcomer to the exoscope. And secondly, it allows the surgeon to maintain an upright and ergonomic position at all times during the operation.^4^

### 2:37 Exoscopic Approach.

During the approach to the trigeminal nerve, the viewing angle has to be changed several times to safely follow the tentorial edge to the cerebellopontine cistern. This can be done quite easily by moving the scope without having the surgeon change his position.

In order to prevent the instruments from being put down frequently, it is advisable to control the focus and zoom using a foot pedal.

### 3:08 Dissection Technique.

The cerebellopontine cisterns are opened and CSF is removed. The suction is used to retract the cerebellum which is protected by a Gelfoam. The arachnoid sheets covering the facial and vestibulocochlear nerve are preserved to prevent any injury.

Conflicting vessels are identified and mobilized.

### 3:48 Treating the Neurovascular Conflict.

Pieces of Teflon are prepared. The little pieces are placed in front of the conflicting vessels and are mobilized under the vessels with a bayonet dissector. It is important to be careful, especially when you manipulate the arteries to prevent vascular injuries to the brainstem. Unfortunately, the very good depth of field that is made possible by the 3D technology cannot be seen in the video. Before the Teflon is fixed with fibrin glue, it is important to check whether the entry site of the nerve root into the brainstem is free of conflicting vessels. A small amount of fibrin glue is used to prevent Teflon dislocation.

Here is another case where a bony prominence of the petrous bone, shown by the asterisk, limits the view of the trigeminal nerve. But here, too, the exoscope offers very good illumination in this narrow corridor and the arterial compression that lies on the back of the nerve root can be relieved.^5^ Therefore, it is almost never the case that an endoscope is needed to provide better visualization, which requires not only expertise in endoscopic interventions but also a significant amount of working space.^6^

In this case, we had to work between the Dandy vein, shown by the white asterisk, and the seventh and eighth cranial nerves, shown by the black asterisk, to reach the trigeminal nerve root.

Here, an optical technology called narrow-band imaging was used. This technology involves the use of interference filters to only use the wavelengths of light that are absorbed by hemoglobin.^7^ This allows for maximum contrast between blood vessels and nerve tissue which might be helpful in depicting smallest vascular structures.

Once again, the vascular-nervous conflict is resolved under the trigeminal root.

### 6:32 Closure Technique.

We perform a multilayer closure of the dura. Gelfoam is placed under the dura and CSF is replaced. The dura is sutured. Mastoid air cells are closed with bone wax, fibrin glue, and pieces of muscle. TachoSil is placed over the dura. The bony defect is replaced by bone cement and attached to the bone with two little titanium plates. Neck muscles, subcutis, and skin are closed in layers.

### 7:06 Conclusions.

Although 3D exoscopes do not change the technique of microvascular decompression, they represent an improvement over existing operation microscopes in terms of ergonomics, resolution, and illumination—essential points for microvascular decompression surgery where the aim is to create the best possible visibility in a narrow corridor.

## Disclosures

The authors report no conflict of interest concerning the materials or methods used in this study or the findings specified in this publication.

## Author Contributions

Primary surgeon: Herta. Assistant surgeon: Dorfer. Editing and drafting the video and abstract: Herta, Dorfer. Critically revising the work: Rössler, Dorfer. Reviewed submitted version of the work: Rössler, Dorfer. Approved the final version of the work on behalf of all authors: Herta. Supervision: Rössler, Dorfer.

## Supplemental Information

### Patient Informed Consent

The necessary patient informed consent was obtained in this study.
